# Genetic Analysis of Influenza A/H1N1pdm Strains Isolated in Bangladesh in Early 2020

**DOI:** 10.3390/tropicalmed7030038

**Published:** 2022-02-28

**Authors:** Abu Hasan, Tadahiro Sasaki, Juthamas Phadungsombat, Ritsuko Koketsu, Rummana Rahim, Nikhat Ara, Suma Mita Biswas, Riku Yonezawa, Emi E. Nakayama, Mizanur Rahman, Tatsuo Shioda

**Affiliations:** 1 Evercare Hospital Dhaka (Ex Apollo Hospitals Dhaka), Dhaka 1229, Bangladesh; rasel.hasan@evercarebd.com (A.H.); rummana.rahim@evercarebd.com (R.R.); nikhat.ara@evercarebd.com (N.A.); suma.biswas@evercarebd.com (S.M.B.); 2 Research Institute of Microbial Diseases, Osaka University, Suita 565-0781, Japan; sasatada@biken.osaka-u.ac.jp (T.S.); juthamas@biken.osaka-u.ac.jp (J.P.); koketsu@biken.osaka-u.ac.jp (R.K.); u148185i@ecs.osaka-u.ac.jp (R.Y.); emien@biken.osaka-u.ac.jp (E.E.N.); 3 Center for Infectious Disease Education and Research, Osaka University, Suita 565-0781, Japan; 4 Mahidol-Osaka Center for Infectious Diseases (MOCID), Faculty of Tropical Medicine, Mahidol University, Bangkok 10400, Thailand

**Keywords:** influenza virus, HA, NA, H1N1pdm09, COVID-19, Bangladesh, evolution

## Abstract

Influenza is one of the most common respiratory virus infections. We analyzed hemagglutinin (HA) and neuraminidase (NA) gene segments of viruses isolated from influenza patients who visited Evercare Hospital Dhaka, Bangladesh, in early 2020 immediately before the coronavirus disease 2019 (COVID-19) pandemic. All of them were influenza virus type A (IAV) H1N1pdm. Sequence analysis of the HA segments of the virus strains isolated from the clinical specimens and the subsequent phylogenic analyses of the obtained sequences revealed that all of the H1N1pdm recent subclades 6B.1A5A + 187V/A, 6B.1A5A + 156K, and 6B.1A5A + 156K with K209M were already present in Bangladesh in January 2020. Molecular clock analysis results suggested that the subclade 6B.1A5A + 156K emerged in Denmark, Australia, or the United States in July 2019, while subclades 6B.1A5A + 187V/A and 6B.1A5A + 156K with K209M emerged in East Asia in April and September 2019, respectively. On the other hand, sequence analysis of NA segments showed that the viruses lacked the H275Y mutation that confers oseltamivir resistance. Since the number of influenza cases in Bangladesh is usually small between November and January, these results indicated that the IAV H1N1pdm had spread extremely rapidly without acquiring oseltamivir resistance during a time of active international flow of people before the COVID-19 pandemic.

## 1. Introduction

Seasonal influenza is one of the most common respiratory virus infections. It is estimated to induce about 3 to 5 million cases of severe illness and about 290,000 to 650,000 respiratory deaths per year prior to the coronavirus disease 2019 (COVID-19) pandemic (WHO fact sheets. Influenza (Seasonal). https://www.who.int/news-room/fact-sheets/detail/influenza-(seasonal); accessed on 19 July 2021). Seasonal influenza is caused by influenza virus, which belongs to the family *Orthomyxoviridae* and is classified into types A, B, and C (International Committee on Taxonomy of Viruses (ICTV). https://talk.ictvonline.org/taxonomy/; accessed on 19 June 2021). Influenza type A (IAV) is the primary type that causes seasonal influenza. It possesses eight negative-sense, single-stranded RNA segments that encode hemagglutinin (HA), neuraminidase (NA), RNA polymerase subunit PB2 (PB2), RNA polymerase subunit PB1 (PB1) and PB1-F2, RNA polymerase subunit PA (PA) and PA-X, two matrix proteins (M1 and M2), two distinct non-structural proteins (NS1 and NEP), and nucleoprotein (NP) [1]. IAVs are classified into subtypes based on their surface glycoprotein antigenicity: there are 18 subtypes of HA and 11 subtypes of NA [2]. HA is a glycoprotein that binds to sialic acid on the host cell surface and induces endocytosis. HA is cleaved into HA1 and HA2 subunits by a protease in the host cell. The HA1 subunit, which is the globular head domain, contains receptor-binding sites for sialic acid, and includes five different antigenic epitopes, i.e., Sa, Sb, Ca (Ca1 or Ca2), and Cb [3,4]. The HA2 subunit, which is the stalk domain, induces fusion between the viral particle and the host cell membrane [5]. Influenza is also known to be a zoonosis, which means that it is transmitted from infected animals, such as avian and swine, to humans [6]. The antigenicity of influenza virus is known to change by antigenic drift due to point mutations in the influenza virus genome and by antigenic shift that is induced by the reassortment of gene segments of two types of influenza viruses that simultaneously infect the same cell in a host [7]. In particular, antigenic shift is known to be a factor in influenza pandemics because it greatly changes the antigenicity of the virus. For example, the genome of the IAV H1N1pdm 2009 strains, which caused a pandemic in 2009 and subsequently became a seasonal flu, encodes PB2 and PA derived from the North American avian lineage, PB1 derived from human seasonal influenza A H3N2, NA and M derived from the Eurasian swine lineage, and HA, NP, and NS derived from the North American classical swine lineage [8]. In addition, antigenic drift is also known to induce mutants that can evade the host immune system through the accumulation of random point mutations in the viral genome due to the lack of proofreading ability of influenza RNA polymerase [7,9]. Influenza virus is evolving year-by-year due to continuous antigenic drift, and as a result, the effectiveness of the influenza vaccine and drug susceptibility of the influenza virus are continuously changing [9]. Therefore, influenza virus surveillance is extremely important for public health. Bangladesh is located between South Asia and Southeast Asia, and it is a location of high cross-border movement of humans and animals [10]. In the present study, we analyzed the HA and NA sequences of viruses isolated from influenza patients who visited Evercare Hospital Dhaka, Bangladesh, in 2020 immediately before the COVID-19 pandemic, caused by severe acute respiratory syndrome coronavirus 2 (SARS-CoV-2), and compared the sequences to those in databases. We found that the recent H1N1pdm subclade that emerged in East Asia in September 2019 had appeared in Bangladesh in January 2020.

## 2. Materials and Methods

### 2.1. Ethics Statement

This study proposal was approved by the Research and Ethics Committee of Evercare Hospital Dhaka (ERC 25/2020-1).

### 2.2. Viruses and Cells

The following virus strains were used as positive controls: A/Suita/112/2011 (H1N1pdm09), A/Suita/20/2007 (seasonal H1N1 before 2009), A/Suita/64/2011 (seasonal H3N2), and B/Suita/1/2011 (type B). Madin–Darby canine kidney (MDCK) cells were used for the propagation of the viruses. The cells were maintained in minimum essential medium supplemented with 10% fetal bovine serum and antibiotics (100 units/mL penicillin, 100 µg/mL streptomycin) in a 5% CO_2_ incubator at 37 °C.

### 2.3. Clinical Specimens

Clinical specimens of nasal or nasopharyngeal fluid were collected at Evercare Hospital Dhaka between January and February 2020 from 94 patients with influenza-like illness as defined by the World Health Organization (WHO) (Global Epidemiological Surveillance Standards for Influenza; https://www.who.int/publications/i/item/9789241506601; accessed on 20 July 2021). Two swabs were taken from each patient: one specimen was tested using the SD BIOLINE influenza antigen test kit (Standard Diagnostics, Inc., Yongin-si, Korea), and the other specimen was inserted into a BD Universal Transport (Becton, Dickinson and Company, Franklin Lakes, NJ, USA) for reverse transcription polymerase chain reaction (RT-PCR) and/or virus isolation. The results of the test kit indicated that there were 21 IVA-positive samples, which were collected from 14 males and seven females with mean age of 24.7 years (range, 0.5–71 years), and no influenza B-positive samples.

### 2.4. Virus Isolation

Influenza virus isolation from clinical specimens was performed as described previously [11]. Briefly, MDCK cells were inoculated with the clinical specimens and incubated at 37 °C in Dulbecco’s Modified Eagle Medium Nutrient Mixture F-12 (Invitrogen, Carlsbad, CA, USA) with 1% Antibiotic-Antimycotic (Invitrogen), 0.4% bovine serum albumin, and 2 μg/mL of Trypsin Acetylated, Type VS from Bovine Pancreas (Sigma, St. Louis, MO, USA) for one week. If no cytopathic effect was observed within one week, up to two more passages were conducted.

### 2.5. RT-PCR for the Classification of Influenza Virus Subtypes in the Clinical Specimens

RT-PCR for the classification of the influenza virus subtypes was performed as described previously [11] with minor modifications. Briefly, viral RNA was extracted from sample specimens using a QIAamp^®^ Viral RNA Mini kit (QIAGEN, Hilden, Germany). RT-PCR was performed with a OneStep RT-PCR kit (QIAGEN) using individual primer sets for H1N1dpm, H1N1 seasonal influenza before 2009, or H3 (Supplementary Table S1). The reaction components for RT-PCR were prepared by mixing 2 μL of 5× QIAGEN OneStep RT-PCR Buffer, 0.4 μL of dNTP mix, 0.2 μL of forward primer (10 μM), 0.2 μL of reverse primer (10 μM), 0.32 μL of QIAGEN OneStep RT-PCR Enzyme Mix, 5.68 μL of Nuclease-free H_2_O, and 1.2 μL of Viral RNA per reaction. The PCR conditions were 50 °C for 30 min and 94 °C for 3 min, followed by 45 cycles of 94 °C for 30 s, 54 °C for 30 s, 72 °C for 30 s, and a final extension at 72 °C for 7 min. The PCR products were subjected to electrophoresis on a 2% agarose gel. RNA extracted from each positive control virus was used as the standard RNA. The concentration of each sample was calculated by fitting the infection titer of each virus to create a standard curve.

### 2.6. Absolute Quantitation of Influenza Viral RNA Using the SYBR Green I Real-Time RT-PCR Assay

Real-time RT-PCR for the quantitation of influenza virus RNA was performed as described previously [12] with minor modifications. Briefly, SYBR green I real-time RT-PCR was performed with a One Step TB Green^®^ Prime ScriptTM RT-PCR kit II (Takara, Shiga, Kusatsu, Japan) and primer sets for the IAV matrix gene (Supplementary Table S1) using Rotor-Gene Q (QIAGEN). The reaction components for RT-PCR were prepared by mixing 6.25 μL of 2 × One Step TB Green RT-PCR Buffer 4, 0.25 μL of forward primer (10 μM), 0.25 μL of reverse primer (10 μM), 0.5 μL of PrimeScript 1step Enzyme Mix 2, 2.75 μL of Nuclease-free H_2_O, and 2.5 μL of Viral RNA per reaction. The real time RT-PCR conditions were: 42 °C for 5 min and 95 °C for 10 s, followed by 43 cycles of 95 °C for 15 s and 60 °C for 1 min, with a melting curve (+0.5 °C every 10 s) at the end.

### 2.7. Sequencing of the HA and NA Genome Segments of IAV H1N1pdm09

The viral RNA was used for cDNA synthesis by the SuperScript TM III First-Strand Synthesis System for RT-PCR (Invitrogen) with Unit 12 primer (AGCAAAGCAGG) as a gene-specific primer [11]. Then, the cDNA was used for PCR amplification by PrimeSTAR^®^ GXL DNA Polymerase (Takara) using amplification primer sets for each target (Supplementary Table S1) [13]. The amplification products were subjected to electrophoresis on a 1.5% agarose gel, and the target DNA bands were purified from the gel using a QIAquick Gel Extraction kit (Qiagen). DNA sequencing was performed by the dideoxynucleotide chain termination method with a BigDyeTM Terminator v3.1 Cycle Sequencing Kit (Applied Biosystems, Foster City, CA, USA) on an Applied BiosystemsTM 3130xl DNA Analyzer (Applied Biosystems). Obtained sequences were deposited in the EpiFlu™ database of the GISAID (www.gisaid.org) (accessed on 21 February 2022) with the accession number EPI1986952, EPI1986953, EPI1986977–EPI1986982.

### 2.8. Sequence Characterization

Phylogenetic analyses were conducted on the HA segment of IAV H1N1pdm09. The IAV H1N1pdm09 sequences were downloaded from the EpiFlu^TM^ database of the Global Initiative on Sharing All Influenza Data (GISAID) database (https://www.gisaid.org/; accessed on 13 September 2021)) [14]. DNA and amino acid sequence analyses were performed with GENETYX^®^ software (GENETYX, Tokyo, Japan) and MEGA X: Molecular Evolutionary Genetics Analysis across computing platforms [15].

### 2.9. Phylogenetic Analysis

The nucleotide sequence dataset was aligned using Muscle in AliView v1.26 [16]. The substitution model was selected, and the maximum likelihood (ML) tree was constructed in IQ-TREE with 1000 ultrafast bootstrap replicates [17]. The time-scaled tree was constructed using the BEAST package with a Bayesian Markov chain Monte Carlo approach [18]. The input dataset was inspected for a positive temporal signal in Tempest v1.5.3 [19], and the nucleotide substitution model was determined by using ModelFinder [20]. An exponential population growth model and the relaxed molecular clock were employed as previously described [21]. Triplicate runs of Markov chain Monte Carlo chain lengths of 30,000,000 generations with sampling every 3000 generations were performed, with individually obtained effective sample sizes over 200 traced in Tracer and combined in LogCombiner. A maximum clade credibility tree was constructed in TreeAnnotator and visualized in Figtree v1.4.4 (http://tree.bio.ed.ac.uk/software/figtree; accessed on 16 October 2021).

## 3. Results

### 3.1. Background of the Clinical Specimens, RT-PCR Analysis, and Virus Isolation

Of 94 patients with influenza-like symptoms, 21 patients were IVA-positive using a rapid diagnosis test kit. There were no influenza B-positive samples. The IVA-positive specimens were collected from 14 males and seven females with mean age of 24.7 years (range, 0.5–71 years), and there was an average of 2.48 ± 1.97 days between the onset of clinical signs and sample collection (Supplementary Table S2). Subsequently, the IVA-positive specimens were subjected to RT-PCR for subtyping and to SYBR green real-time RT-PCR for the quantitation of influenza virus RNA. The subtyping RT-PCR results indicated that 17 of 21 specimens contained H1N1pdm, while the remaining four were all negative for H1N1pdm, seasonal H1N1 before 2009, and seasonal H3N2. Quantitative real-time RT-PCR results indicated that the virus quantity ranged from 0.3 FFU/mL to 4.0 × 10^3^ FFU/mL. The four samples in which we failed to determine subtype showed the lowest, second lowest, third lowest, and fourth lowest virus quantity (range, 0.3–0.5 FFU/mL) on quantitative real-time PCR (Supplementary Table S2). Next, we attempted to isolate the virus from the 17 clinical specimens that were H1N1pdm-positive by RT-PCR subtyping; viruses were successfully isolated from four clinical specimens (No. 10, 15, 46, and 59; Supplementary Table S2). All these isolates were obtained after the second passages.

### 3.2. Genetic Characterization of the HA Segments Obtained in the Present Study

**Figure 1:**
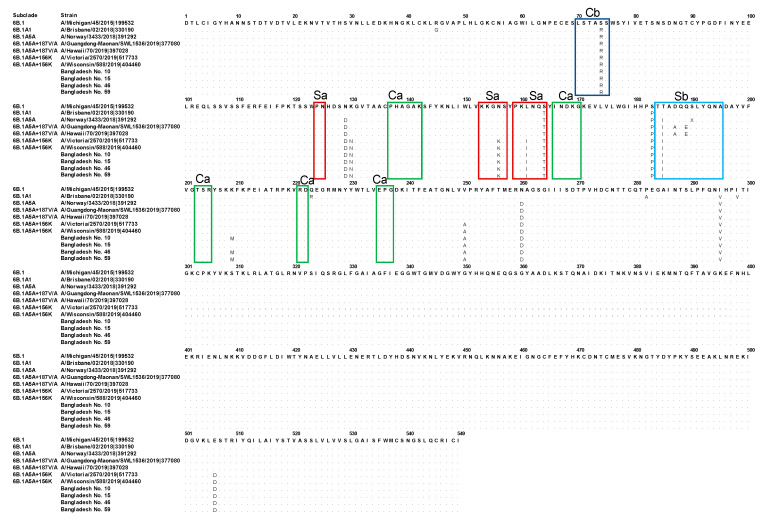
Alignment of the coding amino acid sequences of HA1 antigenic sites in the four strains isolated in Bangladesh in the present study. The coding amino acid sequences of HA1 were aligned to that of the reference strain, A/Michigan/45/2015. The H1N1pdm09 subclades and strain names are indicated on the left side of the figure. The amino acid sequences of each antigenic site are indicated as follows: Sa site (red box), Sb site (light blue box), Ca site (green box), and Cb site (dark blue box).

The four viruses isolated in the present study were subjected to sequencing and phylogenetic analyses. The amino acid sequences of the HA segments were characterized and classified into subclades according to the classification of the February 2019 WHO vaccine composition meeting; we identified seven subclades (6B.1A1–6B.1A7) defined by amino acid substitutions [22]. The sequences of the four isolates were all classified into subclade 6B.1A, as they possessed amino acid substitutions S74R, S164T, and I295V in HA1 as compared to the prototype A/Michigan/45/2015. They were further classified into subclade 6B.1A5A by the presence of the additional amino acid substitutions of S183P, N129D, T185I, and N260D (T185I was in the Sb antigenic site). They also carried K130N, N156K, L161I, V250A, and E506D (N156K and L161I were in the Sa antigenic site), which are specific to subclade 6B.1A5A + 156K (Figure 1, Supplementary Table S3). These results indicated that all the four strains obtained in the present study belonged to this subclade 6B.1A5A + 156K.

### 3.3. Human IAV H1N1pdm09 Detected in Bangladesh from 2018 to 2020

**Figure 2:**
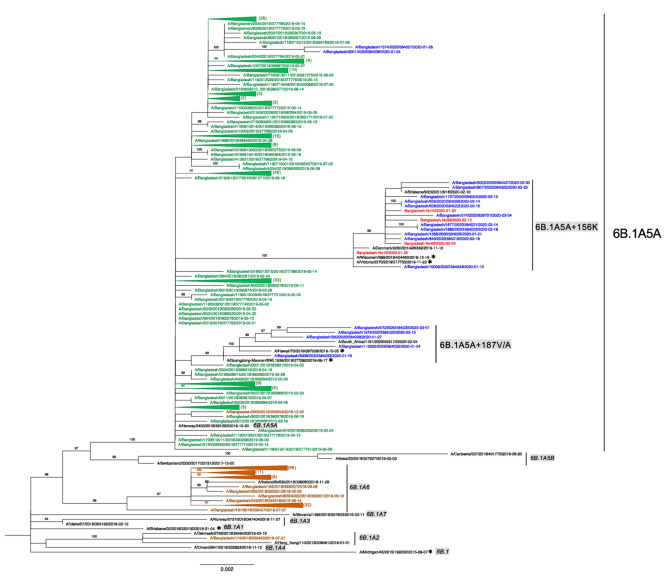
The H1N1pdm09 subclade in Bangladesh in 2018 to 2020. A maximum-likelihood tree of H1N1pdm09 was generated based on the HA gene with TVN + F + G4 and 1000 ultrafast bootstrap replicates using IQ-TREE. The percentage of trees (>80) in which the associated taxa clustered together is shown on the branches. The tree is drawn to scale, with branch lengths being directly proportional to the number of substitutions per site. Nucleotide sequences of the HA genes retrieved from EpiFlu GISAID are labeled as follows: isolate name|isolate ID|collection date. The Bangladesh 2018, 2019, and 2020 sequences are shown in brown, green, and blue, respectively, and the number of collapsed sequences is shown in parentheses at the end. The four sequences obtained in the present study are shown in red. The H1N1pdm09 subclade reference sequences are shown in black, with the subclade names highlighted in gray at the end. Subclade ranges are shown by vertical bars. Asterisks indicate the vaccine strains.

To investigate the subclades that have circulated in Bangladesh in the past, the HA sequences of Bangladesh strains of human IAV H1N1pdm registered in the EpiFlu GISAID database between January 2018 and March 2021 were collected, and a phylogenetic analysis was performed. The sequence dataset consisted of 376 strains (124, 212, and 18 strains registered in 2018, 2019, and 2020, respectively), the four sequences obtained in the present study, and 20 reference strains of 6B.1A and 6B.1A1-A7. Results showed that 122 of 2018 strains were clustered with Ireland/84630/2018, the reference strain of the subclade 6B.1A6 defined by the amino acid substitutions T120A and S183P (Figure 2). The remaining two 2018 strains were classified as either 6B.1A2 or 6B.1A5A (Figure 2, Supplementary Table S3). On the other hand, all of the 2019 strains belonged to subclade 6B.1A5A. Remarkably, the subclade 6B.1A6 that dominated in 2018 had completely disappeared in 2019. Eighteen strains registered in 2020 and the four strains of the present study belonged to subclade 6B.1A5A, which was further phylogenetically classified into three genetic groups: (1) two strains (9.1%) were related to the parental subclade 6B.1A5A strain; (2) five strains (22.7%) belonged to the 6B.1A5A + 187V/A subclade with the vaccine viruses of Guangdong–Maonan/SWL1536/2019 and Hawaii/70/2019 carrying the specific amino acid mutations of D187V/A and Q189E; and (3) 15 strains (68.2%), including the four strains obtained in the present study, belonged to the 6B.1A5A + 156K subclade with the vaccine viruses Wisconsin/588/2019 and Victoria/2570/2019 with N156K [23]. These results indicated that another subclade 6B.1A5A + 187V/A was also present in Bangladesh in 2020 as a minor population.

### 3.4. Evolution of Subclades 6B.1A5A + 187V/A and 6B.1A5A + 156K of Bangladesh 2020 Human IAV H1N1pdm09

**Figure 3:**
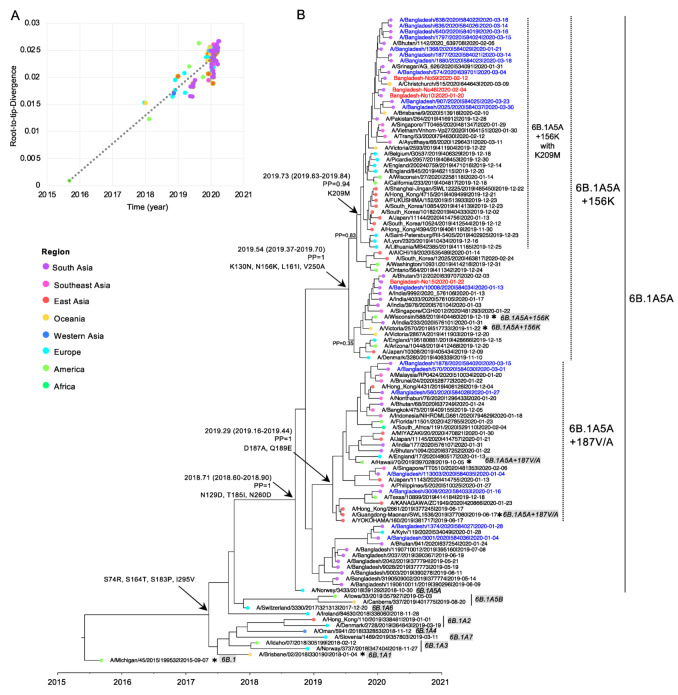
Evolution of Bangladesh H1N1pdm subclade 6B.1A5A strain 2020. (**A**) A root-to-tip analysis of the dataset. (**B**) The maximum clade credibility (MCC) tree based on the HA gene of H1N1pdm was estimated in BEAST package v1.10.4 with HKY + F + I, an exponential population growth model, and the relaxed molecular clock. The nucleotide sequences of the HA genes retrieved from EpiFlu GISAID are labeled as follows: isolate name|isolate ID|collection date (yyyy-mm-dd). The H1N1pdm strains, including the H1N1pdm subclade reference strains, are shown in black, with the clade name highlighted in gray at the end. Ranges of subclades 6B.1A1, 6B.1A2, 6B.1A3, 6B.1A4, 6B.1A5A, 6B.1A5B, 6B.1A6A, and 6B.1A7A are shown by vertical bars. Ranges of 6B.1A5A + 156K, 6B.1A5A + 187V/A, and 6B.1A5A + 156K with K209M are shown by vertical doted bars. Asterisks indicate the vaccine strains. The Bangladesh 2020 strains in EpiFlu and the newly obtained Bangladesh 2020 sequences in the present study are shown in blue and red, respectively. The regions in which the strains circulated are indicated at the branch tips and are color-coded as indicated in the legend on the left. The x-axis of the tree indicates the time (year). The tMRCA and interval, posterior probability (PP), and the occurrence of amino acid mutations of the key nodes are indicated by arrows.

To examine the origin, divergence time, and related strains of these three genetic groups of Bangladesh 2020 strains, a new dataset was prepared for Bayesian phylogenetic analysis; the dataset included the most related strains obtained from a BLAST analysis conducted in EpiFlu GISAID, the earlier strains of 6B.1A5A + 187V/A and 6B.1A5A + 156K in influenza genomic epidemiology provided by Nextstrain in GISAID [24], and the reference strains of 6B.1A and 6B.1A1–A7. In this new dataset, the correlation coefficient between the collection date and root-to-tip divergence was 0.725 (Figure 3A). The phylogenetic tree (Figure 3B) revealed that Bangladesh 2020 subclade 6B.1A5A descended from Bangladesh 2019 and was related to strains from Bhutan and Kyiv with similar collection dates. Five of the Bangladesh 2020 6B.1A5A + 187V/A strains had the same time to the most recent common ancestor (tMRCA) of 2019.29 (posterior probability (PP) = 1) and were related to the Texas and Hong Kong strains collected in December 2019 and the Japan, Malaysia, and Brunei strains collected in January 2020. All of the strains in the 6B.1A5A + 187V/A cluster had the two amino acid substitutions of D187A and Q189E. On the other hand, 15 of Bangladesh 2020 6B.1A5A + 156K strains fell into the 6B.1A5A + 156K cluster carrying K130N, N156K, L161I, and V250A with a tMRCA of 2019.54 (PP = 1). Within this cluster, Bangladesh 2020 strains were observed in two clades with a PP of 0.35 and 0.83: (1) two strains, including No. 15 in the present study that was related to the Bhutan and India strains collected in January and February 2020 with the aforementioned mutations, and (2) 13 strains, including No. 10, 46, and 50 in the present study that were related to the Brisbane, Christchurch, Srinagar, and Bhutan strains collected in January and February 2020. An additional substitution of K209M was detected in this subclade, and the strains that carried K209M formed a distinct subclade with tMRCA of 2019.73 (PP = 0.94). These results indicated that all the human IAV H1N1pdm09 subclades that dominated in Bangladesh in 2020 emerged within a year in other countries.

### 3.5. Global Circulation of Subclades 6B.1A5A + 187A and 6B.1A5A + 156K

To clarify the circulation of subclades 6B.1A5A + 187V/A and 6B.1A5A + 156K of 6B.1A5A around the world after March 2020, we collected all of the data on human H1N1 HA in GISAID from March 2020 to March 2021. Subsequently, a total of 778 sequences (774 sequences from GISAID and the four sequences obtain in the present study) were used to determine the proportion of subclades 6B.1A5A + 187V/A and 6B.1A5A + 156K among the viruses according to the previously mentioned clade-specific amino acid substitutions. The results showed that 756 of the total 778 sequences (97.17%) were 6B.1A. Among them, 733 sequences were subclade 6B.1A5A (96.95% of subclade 6B.1A and 94.21% of all H1N1 strains), in which 276 sequences were subclade 6B.1A5A + 187V/A (37.65% of subclade 6B.1A5A and 38.4% of all H1N1 strains), and 358 were subclade 6B.1A5A + 156K (48.84% of subclade 6B.1A5A and 46.01% of all H1N1 strains). In addition, 136 sequences contained K209M (37.99% of subclade 6B.1A5A + 156K and 17.48% of all H1N1 strains; Supplementary Table S4). These results indicated that the human IAV H1N1pdm09 subclades that dominated in Bangladesh in 2020 also dominated all over the world.

### 3.6. Genetic Characterization of NA Segments Obtained in the Present Study

NA segment sequences of Bangladesh strains of human IAV H1N1pdm registered in the EpiFlu GISAID database between January 2018 and March 2021 were collected, and a phylogenetic analysis was performed. The sequence dataset consisted of 356 strains (129, 209, and 18 strains registered in 2018, 2019, and 2020, respectively) and the four sequences obtained in the present study (Supplementary Table S4). The results showed almost the same pattern as HA segments (Supplementary Figure S1). Nearly all the strains in 2018 (126 out of 129 strains) formed a distinct cluster from the rest of strains. This result indicated that NA sequences that dominated in 2018 totally disappeared from Bangladesh in 2019 and 2020 as observed in HA sequences. Furthermore, the strains collected in 2020 formed three distinct clusters, just like HA genes. NA segment analysis indicated that all four sequences from the present study lacked the H275Y amino acid substitution (Supplementary Table S5) that confers oseltamivir resistance [25]. The H275Y mutation was found only one strain (A/Bangladesh/4005 2019|EPI ISL 395161 NA) in the Bangladesh strains registered in the GISAID database between January 2018 and March 2021 (Supplementary Table S5); however, it was found in seven (five 6B.1A5A + 156K, one 6B.1A5A + 187V/A, and one non-6B clade) of the 737 strains registered from all over the world between March 2020 and March 2021 (Supplementary Table S6). These results indicated that oseltamivir resistance was still rare in these human IAV H1N1pdm09 subclades.

## 4. Discussion

In the present study, we determined the nucleotide sequences of HA and NA genome segments of IAV H1N1pdm specimens collected in Bangladesh between January and February 2020. The HA gene sequences were then compared to the sequences in EpiFlu GISAID database. The viruses obtained in the present study belonged to subclade 6B.1A5A + 156K. Our molecular clock analysis suggested that another subclade, 6B.1A5A + 187V/A, which was also present in Bangladesh in 2020, emerged in April 2019 (interval: March to June 2019; 2019.29, HPD:2019.16–2019.44) likely in East Asia since this subclade was first detected in June 2019 in China, Japan, and Hong Kong. On the other hand, 6B.1A5A + 156K was suggested to have emerged slightly later in July 2019 (interval: May to September 2019; 2019.54, HPD:2019.37-2019.70), and it likely originated in Australia, the United States, or Denmark, where the first viruses were detected in November 2019. Subsequently, these two subclades spread worldwide, particularly in America, East Asia, South Asia, and Southeast Asia. Notably, the more recent clade 6B.1A5A + 156K with K209M was also observed, and its time of emergence was inferred to be September 2019 (August to November 2019). This virus was detected for the first time in Hong Kong and South Korea in late November and early December 2019, and it later became dominant in Bangladesh, especially in March 2020. Therefore, the present study showed that all of the recent subclades of H1N1pdm, 6B.1A5A + 187V/A, 6B.1A5A + 156K, and 6B.1A5A + 156K with K209M, all of which first emerged between April and September 2019 in countries other than Bangladesh, were also present in Bangladesh in January 2020. Since the number of influenza cases in Bangladesh is usually small between November and January, [26], these results demonstrated that the IAV had spread extremely rapidly.

The amino acid substitution of N156K in subclade 6B.1A5A + 156K is reported to have a significant impact on vaccine effectiveness. Only 2 years after the 2009 H1N1pdm pandemic, Strengell et al. showed that the N156K mutation had reduced the effectiveness of the influenza vaccine [27]. Then, in 2013, Guarnaccia et al. showed that the N156K mutation altered the binding efficiency of HA-specific antibodies [28]. However, the prevalence of the N156K mutation was as low as 0.15% among the global isolates in registered databases in 2009 to 2012 [28] and was 8.2% among Vietnamese isolates in 2010 to 2013 [29]. On the other hand, the results of the present study showed that in Bangladesh, 0%, 1.9%, and 60.9% of the isolates had this mutation in 2018, 2019, and 2020, respectively, and after March 2020, 47.7% of the isolates reported worldwide had this mutation. These results indicated that the prevalence of this mutation has rapidly increased since 2020. Tenford et al. reported that the sera from individuals vaccinated with the 2019–2020 season vaccine recognized the 6B.1A5A + 156K strain poorly, and the vaccine failed to protect against infection by this virus [30]. Therefore, the WHO has changed their recommendation on the composition of the influenza vaccines for the 2021 southern hemisphere influenza season and the 2021–2022 northern hemisphere influenza season to vaccines using the A/Victoria/2570/2019 strain or A/Wisconsin/588/2019 strain as reference strains for H1N1 [23,31]. Vaccines using these strains showed better recognition of 6B.1A5A + 156K but poor recognition of other strains, such as 6B.1A5A + 187V/A, which lacks the N156K mutation [23,30]. However, 6B.1A5A + 187V/A strains have also been detected in many cases since 2020; therefore, monitoring of these strains may be important for assessing the effectiveness of future vaccines.

COVID-19 is a respiratory infection similar to influenza. The preventative measures that have been adopted against the spread of COVID-19 are thought to have also reduced the number of influenza cases [23]. In addition, asymptomatic patients play an important role in influenza transmission [32,33], and a decrease in the total number of influenza patients also decreases the number of asymptomatic patients, which may result in a reduction in the herd immunity to influenza viruses. Due to the shift of human and equipment resources to COVID-19 testing, there has been a reduction of influenza surveillance and reporting activities, and the number of influenza virus strains being registered in databases recently has greatly decreased when compared to previous years [23]. Although it remains unclear how SARS-CoV-2 infection affects influenza virus infection, several cases of co-infection with both of these viruses have been reported [34]. Therefore, it is expected that influenza surveillance will become even more important in the future [35].

## 5. Conclusions

We determined nucleotide sequences of HA and NA genes of IAV H1N1pdm specimens collected in Bangladesh between January and February 2020. Phylogenic analyses revealed that all of three H1N1pdm recent subclades were already present in Bangladesh in January 2020. Molecular clock analyses suggested that these subclade emerged in April-September 2019 in countries other than Bangladesh. These results indicated that the IAV H1N1pdm had spread extremely rapidly during a time of active international flow of people before the COVID-19 pandemic.

## Data Availability

The data presented in this study are available in this article and Supplementary Materials.

## Supplementary Materials

The following supporting information can be downloaded at: www.mdpi.com/article/10.3390/tropicalmed7030038/s1, Table S1: Primers used for RT-PCR subtyping, real-time PCR, cDNA synthesis, and sequencing; Table S2: Data on the clinical specimens and the immunochromatographic test, RT-PCR subtyping, quantitative real-time RT-PCR, and virus isolation results; Table S3: Amino acid sequences of HA of the Bangladeshi IAVs registered from 2018 to 2020; Table S4: Amino acid sequences of HA of the global IAVs registered from March 2020 to March 2021; Table S5: Amino acid sequences of NA of the Bangladeshi IAVs registered from 2018 to 2020; Table S6: Amino acid sequences of NA of the global IAVs registered from March 2020 to March 2021; Figure S1: The H1N1pdm09 NA genes in Bangladesh in 2018 to 2020. A maximum-likelihood tree of H1N1pdm09 was generated based on the NA gene with 1000 ultrafast bootstrap replicates using IQ-TREE. Nucleotide sequences of the NA genes retrieved from EpiFlu GISAID are labeled as follows: isolate name|isolate ID. The Bangladesh 2018, 2019, and 2020 sequences are shown as brown circles, green squares, and blue rhombuses, respectively. The four sequences obtained in the present study are shown as red rhombuses. The H1N1pdm09 reference sequences are shown as black triangles. The strain with H275Y amino acid substitution is shown as a right green square.
