# Supplementary material for: Genetic Analysis of Influenza A/H1N1pdm Strains Isolated in Bangladesh in Early 2020
Source: Trop Med Infect Dis. 2022 Feb 28;7(3):38. doi: 10.3390/tropicalmed7030038 (PMC8949303; doi:10.3390/tropicalmed7030038)
Supplement: Supplementary file 1 [file tropicalmed-07-00038-s001.zip › Supplementary Figure S1.pdf]

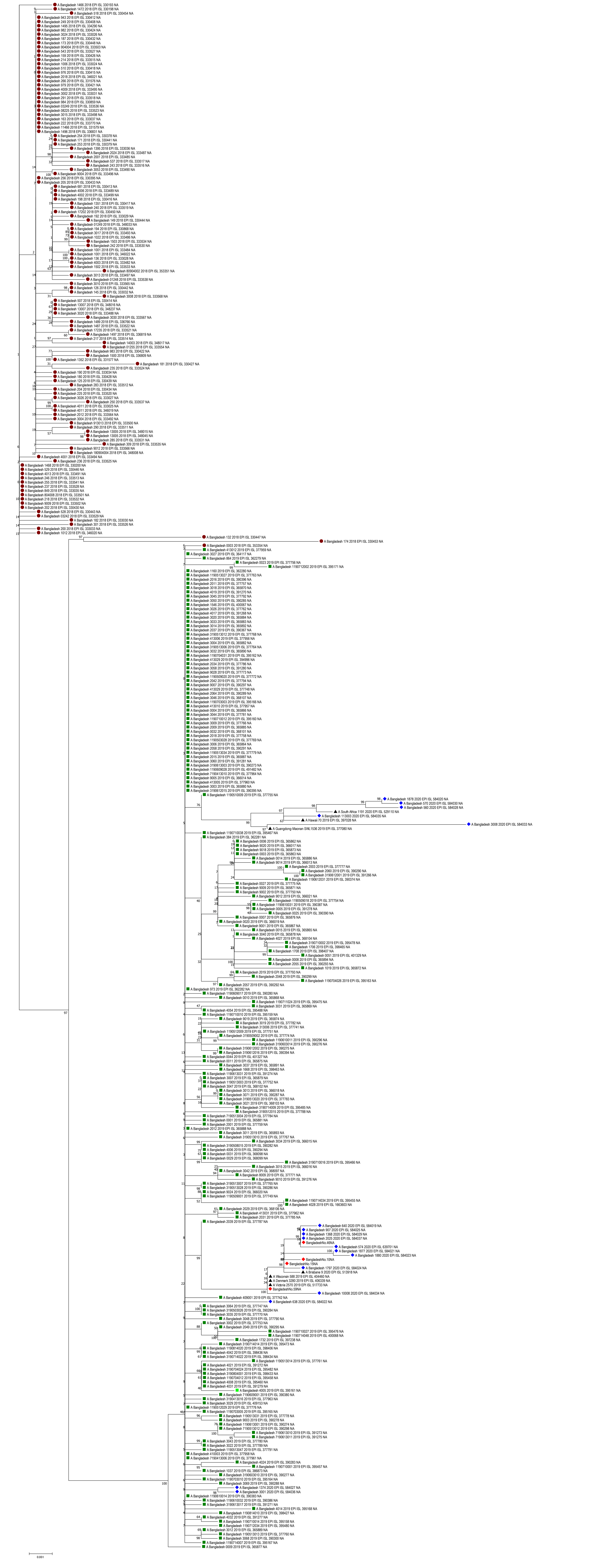

**Supplementary Figure 1. The H1N1pdm09 NA genes in Bangladesh in 2018 to 2020.** A maximum-likelihood tree of H1N1pdm09 was generated based on the NA gene with 1,000 ultrafast bootstrap replicates using IQ-TREE. Nucleotide sequences of the NA genes retrieved from EpiFlu GISAID are labeled as follows: isolate name|isolate ID. The Bangladesh 2018, 2019, and 2020 sequences are shown as brown circles, green squares, and blue rhombuses, respectively. The four sequences obtained in the present study are shown as red rhombuses. The H1N1pdm09 reference sequences are shown as black triangles. The strain with H275Y amino acid substitution is shown as a right green square.
